# Manno-oligosaccharides as a promising antimicrobial strategy: pathogen inhibition and synergistic effects with antibiotics

**DOI:** 10.3389/fmicb.2025.1529081

**Published:** 2025-03-24

**Authors:** Rachel E. Asbury, Bradley A. Saville

**Affiliations:** ^1^Bioprocess and Enzyme Technology Lab, Department of Chemical Engineering and Applied Chemistry, University of Toronto, Toronto, ON, Canada; ^2^College of Dietitians of Ontario, Toronto, ON, Canada

**Keywords:** manno-oligosaccharides, pathogens, antimicrobial resistance, antibiotic potentiation, carbohydrate structure–function

## Abstract

Infections caused by pathogenic bacteria pose a significant health challenge to humans and animals, especially given the rising incidence of antimicrobial resistance. Addressing this challenge has resulted in initiatives seeking alternatives to traditional antibiotics. Manno-oligosaccharides (MOS) exhibit pathogen-binding properties, due to their ability to prevent bacterial adhesion to epithelial cells, such as those within the urinary tract and intestinal epithelium. This suggests that MOS could offer a promising alternative to antibiotics. In this study, we explore the ability of various *β*-MOS products to inhibit the growth of *Escherichia coli, Klebsiella pneumoniae, Listeria monocytogenes,* and *Streptococcus mutans*, in addition to their ability to render antibiotics more effective. Inhibition profiles were distinct for each bacterial strain and differed according to *β*-MOS structure. Antibiotics were significantly potentiated by MOS in some cases, such as ceftazidime against *K. pneumoniae*. This research shows the role of carbohydrate structure in the anti-bacterial properties of non-digestible oligosaccharides such as MOS and positions MOS as a promising strategy in the treatment of bacterial infections.

## 1 Introduction

Pathogenic bacterial infections are a global health challenge given the rising incidence of antimicrobial resistance (AMR), and at least 1.27 million deaths yearly are directly attributable to bacterial AMR (Murray et al., 2022). In what is known as the “post-antibiotic era,” alternatives to conventional antibiotics that effectively treat bacterial infections, including those caused by resistant strains, are urgently needed. Next-generation antimicrobial treatments may involve alternatives to antibiotics or antibiotic adjuvant therapies, of which carbohydrate-based non-digestible oligosaccharides (NDOs) may be promising candidates due to their broad antimicrobial abilities (Asadpoor et al., 2020).

**Figure 1:**
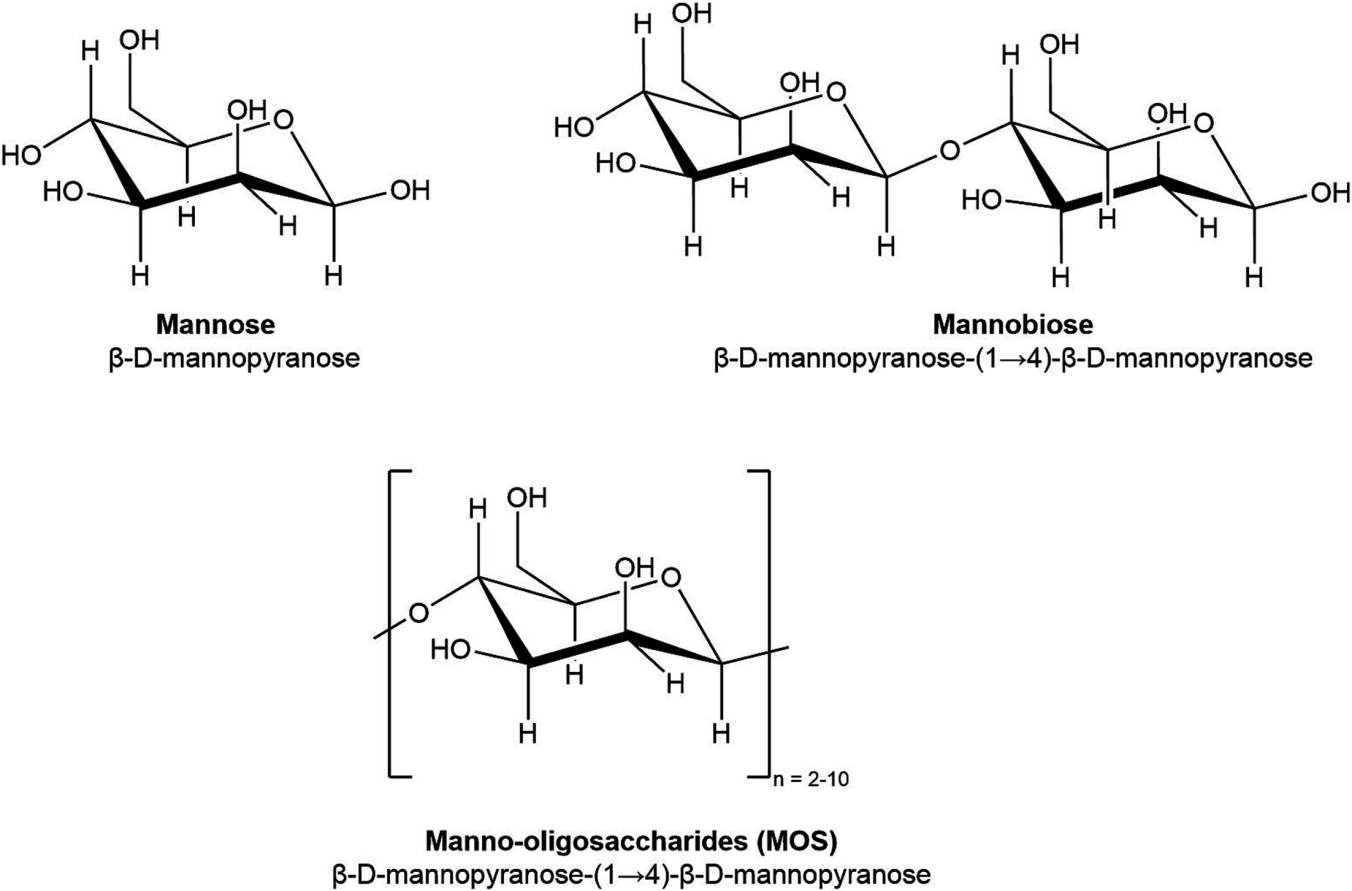
Chemical structure of mannose (DP 1), mannobiose (DP 2), and manno-oligosaccharides (DP 2–10).

Manno-oligosaccharides (MOS) are one category of NDOs that are of particular interest due to their demonstrated abilities to reduce bacterial loads *in vitro* and *in vivo* for a range of pathogens (Spring et al., 2015). As oligosaccharides, MOS are medium-length carbohydrate chains, which are composed of 2–10 monomers of mannose (Figure 1). Yeast-derived *α*-MOS are characterized by mannose subunits linked by *α*-(1-3) or (1-6) glycosidic linkages, while hemicellulose-derived *β*-MOS from sources such as guar gum, coffee grounds, and coconut byproducts are composed of mannose linked by *β*-(1-4) glycosidic linkages (Jana et al., 2021; Saville and Saville, 2020).

In the first stage of a bacterial infection, type-1-fimbriated enteropathogens bind to mannose sequences on the surfaces of host cells and recruit other bacteria once adhered (Firon et al., 1987; Firon et al., 1983; Ribet and Cossart, 2015). The type-1 fimbriae are a category of bacterial adhesins possessed by enteropathogens such as *K. pneumoniae* and *E. coli* with a particularly high affinity for mannose, and binding of fimbriae to host cells can be blocked by mannose and mannose-based oligomers in a dose-dependent manner (Knight and Bouckaert, 2009). This mannose-mediated binding has encouraged further study into anti-adhesion therapies using D-mannose and MOS, such as in the treatment of urinary tract infections (Kranjčec et al., 2014; Lenger et al., 2020).

In addition to the mannose-mediated binding of fimbriated members of the Enterobacteriaceae family, MOS have been shown to reduce the growth of non-fimbriated bacteria such as gram-positive *Clostridia* spp. (Spring et al., 2015), *S. aureus* (Pangsri et al., 2015), and *L. monocytogenes* (Shubhashini et al., 2022). Other NDOs such as alginate oligosaccharides, chito-oligosaccharides, and human milk oligosaccharides have been reported to interact directly with the surface of bacterial cells. This interaction reduces the ability of bacteria to form biofilms and hinders nutrient flows, and promotes the disintegration of bacterial cell membranes (Asadpoor et al., 2021a; Craft et al., 2018a; Lu et al., 2019). In addition, some research has suggested that oligosaccharides can increase the effectiveness of antibiotics by increasing bacterial membrane permeability, promoting antibiotic uptake, and resulting in a lower effective dose (Craft et al., 2018b; Yang et al., 2017). The concept of antibiotic adjuvant therapy, in which substances that potentiate the action of antibiotics are co-administered with them, is being revisited in the context of AMR concerns (Paul et al., 2023).

Differences in carbohydrate structure appear to influence the effectiveness of MOS in inhibiting pathogens. In one study, while both *α*-MOS and *β*-MOS inhibited the tested pathogens to some degree, *α*-MOS was superior in inhibiting the growth of pathogens *S. aureus*, *S. dysenteriae,* and *S. enterica*, while *β*-MOS resulted in better inhibition of *E. coli* and *S. enteritidis* (Pangsri et al., 2015). However, this is an understudied area of research, and other structural factors beyond the *α*- and *β*-MOS distinction, such as degree of polymerization (DP), are likely to affect differences in inhibition abilities of MOS, consistent with findings for other anti-pathogenic NDOs (Asadpoor et al., 2020).

Carbohydrates and glycans play a crucial biological role in bacteria, mediating binding events between pathogens and their hosts, participating in glycosylation and cell signaling, and serving as structural components of biofilms (Day et al., 2015; Sauer et al., 2019). These interactions make NDOs important targets in treating and preventing bacterial infections. Additionally, oligosaccharides do not pose the same risk of AMR. For example, the primary mechanisms by which MOS inhibit pathogens are related to interactions with highly conserved bacterial structures, such as cell walls and fimbriae (Asadpoor et al., 2020; Knight and Bouckaert, 2009; Kolenda et al., 2019; Stahlhut et al., 2012). In addition to the direct effects of MOS against various bacterial strains, MOS have also been shown to indirectly influence pathogen colonization within microbial communities by selectively promoting the growth of beneficial or commensal bacteria that compete with pathogens for resources, demonstrating a “prebiotic” effect (Jana et al., 2021; Prayoonthien et al., 2019; Sathitkowitchai et al., 2021).

This study aims to evaluate the ability of hemicellulose-derived *β*-MOS to enhance antibiotic effectiveness and inhibit the growth of bacterial strains *Escherichia coli, Klebsiella pneumoniae, Listeria monocytogenes* and *Streptococcus mutans*. Furthermore, we seek to explore structure–function relationships by comparing the inhibitory properties of two MOS products with distinct structural compositions.

## 2 Materials and methods

### 2.1 Production of manno-oligosaccharides by the hydrolysis of copra meal

Manno-oligosaccharides (MOS) were produced by the enzymatic hydrolysis of copra meal, a mannan-rich byproduct from coconut processing, as previously described by this research group (Di Pede et al., 2024). Two distinct MOS products were produced: low-molecular-weight MOS (LMW-MOS) in an offsite manufacturing plant and high molecular weight (HMW-MOS) in a 4 L batch process in-house. The HMW-MOS was produced using a shorter reaction time, resulting in less hydrolysis of mannan and a product with a greater proportion of oligomers in the higher-weight fractions. The reaction conditions for LMW-MOS resulted in a greater proportion of oligomers in the shorter DP fractions. The process for LMW-MOS included a spray-drying step, so LMW-MOS was dissolved in MilliQ water for all experiments and analyses. The concentrations of LMW-MOS and HMW-MOS were matched for all growth assays on a dry weight basis by assessing % solids (w/v) of LMW-MOS and HMW-MOS using an HE53 Moisture Analyzer (Mettler Toledo) and diluting liquid HMW-MOS to the equivalent solids % of LMW-MOS for each target concentration. A single batch of product was used for all experiments using LMW-MOS and HMW-MOS.

### 2.2 Structural characterization of MOS

The oligomer and monomer composition of both LMW-MOS and HMW-MOS was determined using high-performance liquid chromatography (HPLC) and liquid chromatography–mass spectrometry (LCMS). The composition of mannose, glucose, galactose, xylose, arabinose, and cellobiose of both LMW-MOS and HMW-MOS was measured by HPLC using an Aminex HPX-87H column (Bio-Rad) with a column temperature of 80°C, a refractive index detector (55°C), a mobile phase of water with a flow rate of 0.6 mL/min, and an injection volume of 20 μL. Monomer and cellobiose standards were purchased from Sigma-Aldrich. To quantify the proportion of LMW-MOS and HMW-MOS in the oligomer fraction, liquid characterization was performed according to methods described elsewhere (Sluiter et al., 2008). Briefly, samples of LMW-MOS and HMW-MOS were hydrolyzed with 70% sulfuric acid (Sigma-Aldrich) and autoclaved at 121°C for 1 h to break down all carbohydrate polymers and oligomers into monomers. Hydrolyzed samples were analyzed using HPLC, and the total oligomers were quantified as the difference in monomers between the hydrolyzed and non-hydrolyzed samples, adjusted for differences in molecular weight due to the addition of water during hydrolysis.

The composition of mannose oligomers of DP 2-6 (mannobiose, mannotriose, mannotetraose, and mannohexaose) in both MOS types was determined using LCMS due to overlapping peaks and inconsistent separation of oligomers on our HPLC system. A UHPLC–MS system was used with an Aminex HPX-87H column (Bio-Rad) with a mobile phase of 0.1% formic acid and a heated electrospray ionization probe (HESI-II). Oligomer standards (mannobiose, mannotriose, mannotetraose, and mannohexaose) were purchased from Megazyme. Quantification of both monomers and oligomers was performed using a series of standards at various concentrations to create a standard curve. The % solids of both LMW-MOS and HMW-MOS were determined using an HE53 Moisture Analyzer (Mettler Toledo) to quantify monomers and oligomers on a % dry matter (DM) basis.

### 2.3 Bacterial strains and culturing conditions

Bacterial strains *Klebsiella pneumoniae* strain 1101160, *Listeria monocytogenes* strain 1071/53, and *Streptococcus mutans* strain NCTC 10449 were supplied by ATCC (Cedarlane). Strain *E. coli K12* MG 1655 was obtained from the Mahadevan lab (BioZone, University of Toronto). LB Lennox media (BioShop) was used for growing liquid cultures of *E. coli*, while *K. pneumoniae* was grown in TSB media (MilliporeSigma), and *L. monocytogenes* and *S. mutans* were cultured with BHI media (MP Biomedicals). All media was purchased as pre-formulated, dehydrated media prepared according to the manufacturer’s instructions. All media was heat sterilized with autoclaving before it was used in culturing experiments. For culturing on agar plates, liquid media was formulated with 2% agar, autoclaved, and then poured aseptically into plates. All bacterial strains were maintained on 30% glycerol stocks at −80°C. Cultures were revived by inoculating 1 μL of frozen bacterial stock into 3–5 mL of autoclaved media and incubating at 37°C overnight under shaking, aerobic conditions. To obtain isolates, liquid cultures were streaked on agar plates of their corresponding media and incubated at 24-48 h at 37° C. All plates were stored at 4° C until being used in growth studies.

### 2.4 Substrates for bacterial assays

Stock solutions of LMW-MOS and HMW-MOS for all bacteria assays were prepared at equal concentrations as described in section 2.2. For the growth inhibition and antibiotic potentiation assays, 100 mg/mL stocks of LMW-MOS and HMW-MOS were prepared, and 100 mg/mL stock of mannose was prepared by dissolving dry powder in MilliQ water. For the growth inhibition assays using individual MOS oligomers, 50 mg/mL solutions of mannobiose, mannotriose, and a 1:1 combination of mannotetraose and mannotriose were prepared by dissolving dry powders in MilliQ water. All substrates were filter-sterilized using a 0.22-μm syringe filter before being used in culturing experiments.

### 2.5 Growth inhibition assay using the broth microdilution method

The broth microdilution method is a technique used to determine the minimum inhibitory concentration (MIC), the lowest concentration of a compound that inhibits the visible growth of bacteria under controlled conditions (Wiegand et al., 2008). While MICs have been reported for various types of oligosaccharides (Asadpoor et al., 2021b), complete inhibition is not commonly observed with sugar substrates. This is likely due to the ability of some bacteria to utilize them as a carbon source and the fact that their method of inhibiting bacterial growth tends to be bacteriostatic rather than bactericidal (Mizzi et al., 2020). Nonetheless, the broth microdilution method is an effective tool for screening the inhibitory ability of oligosaccharides at various concentrations.

Aseptic conditions were maintained for all culturing experiments by working next to a Bunsen burner. Working cultures of bacteria were obtained by inoculating one isolate colony from agar stocks using a flame-sterilized inoculating loop into 3–5 mL of prepared liquid media. The cultures were incubated at 37°C overnight under shaking, aerobic conditions. Each culture was diluted to OD 0.01 with fresh media.

A sample well plate design is included in the Supplementary material. Within a 96-well polystyrene well plate (Falcon), 90 μL of sterile media was added to each well, and then 90 μL of the 100 mg/mL stock solution of carbon substrate (LMW-MOS, HMW-MOS, or mannose) was added to the first column and serially diluted across each row using a multi-channel pipette, following the previously outlined protocol (Wiegand et al., 2008). To each well, 10 μL of bacterial culture was added to reach a final concentration of roughly 1.0 × 10^5^ CFU/mL. The plate was then incubated for 18–24 h under aerobic, shaking conditions and bacterial growth was measured continuously at OD600 using a microplate reader (Tecan Infinite 200 Pro). A blank reading of the plate was taken before adding bacterial cultures, and the blank reading was subtracted from each subsequent reading for the growth analysis. The media alone was used as the growth control, and the media alone without cultures added was used as a sterility control.

### 2.6 Antibiotic preparation and determination of MICs

Antibiotics with a range of mechanisms of action against bacteria were selected for the antibiotic sensitization studies, using methods previously described (Craft et al., 2018b). Antibiotics tested included cell wall synthesis-inhibiting penicillin G (Cayman Chemical) and ceftazidime hydrate (Cayman Chemical), and protein synthesis-inhibiting clindamycin (Cayman Chemical) and gentamicin sulfate (AdipoGen). Stock solutions of antibiotics were prepared in a two-step dilution: penicillin and ceftazidime were prepared by dissolving powder in PBS and then diluting with PBS to the target concentration; clindamycin was prepared by dissolving powder in 90% ethanol and then diluting with PBS to the target concentration; and gentamicin was prepared by dissolving powder in water and diluting with water to the target concentration. All antibiotics were filter-sterilized before use in bacterial assays.

Each antibiotic was first screened against all four pathogen types to determine the minimum inhibitory concentration (MIC) using the broth microdilution method as described above, with some modifications. Working cultures of bacteria were obtained by inoculating one isolate colony from the agar plate aseptically into 3–5 mL of prepared liquid media. The cultures were incubated overnight at 37°C under shaking, aerobic conditions, and then diluted to OD_600_ = 0.1 with fresh media the following morning. Antibiotics were prepared at twice the highest target concentration and then serially diluted within the well plate. Each well was inoculated with 10 μL of bacterial culture. The plates were then sealed with breathable plate seals and incubated aerobically at 37°C under shaking conditions (200 RPM). After 24 h, bacterial growth was measured as OD_600_ using a microplate reader (Tecan Infinite 200 Pro). The MIC was defined as the lowest concentration at which no growth was observed, as measured by turbidity at OD_600_.

### 2.7 Bradford assay to measure protein concentration

Antibiotics with trends toward potentiation by MOS were used for further assays to measure the ability of MOS to increase protein concentration in the bacterial culture supernatant, adapted from methods described previously (Kruger, 2009; Moghimi et al., 2016). Starting cultures of all bacterial strains were grown within sterile media overnight to reach OD_600_ = 1.0. Bacterial cells were washed by pelleting at 4000 g, removing the supernatant, adding sterile PBS (pH 7.2, Gibco), and then gently vortexing. The cells were washed three times with PBS to remove culturing media and then re-suspended in sterile PBS to reach a final concentration of OD_600_ = 0.5. Stock solutions of MOS were prepared at 2.5x the target concentration, and antibiotics were prepared at 10x the target concentration. Within 96-well plates, 50 μL of bacterial culture was combined with 40 μL of MOS solution and 10 μL of antibiotics and then incubated under shaking conditions at 37°C for 1 h. The cultures were incubated under their respective MOS and antibiotic conditions for 1 h to assess protein concentration while limiting culture growth on the substrate. After 1 h, the plates were centrifuged in an Avanti J-E centrifuge (Beckman Coulter) at 4000 g for 5 min to pellet cells. A 10 μL aliquot of the supernatant was pipetted into a fresh well plate, and 200 μL of Bradford reagent (BioRad) was added along with standards of bovine serum albumin (BSA). After 15 min, the OD_595_ was measured using a plate spectrophotometer, and protein concentration was quantified using a standard curve developed with BSA standards.

### 2.8 Statistical and data analysis

Data are reported as the mean values ± standard error (SEM) of at least three independent experiments routinely performed in triplicate. The experimental AUC of growth curves was calculated using the R package growthcurver (Sprouffske, 2020). Significant differences in the AUC or final OD_600_ between groups were determined using Welch’s two-sample independent *t*-test, and *p*-values were adjusted using Bonferroni *post-hoc* adjustments for multiple comparisons. Statistical significance was calculated using the R package rstatix (Kassambara, 2023). Chemical structures were produced using ChemDraw (ChemDraw Professional, 2022). Figures were made using the R package ggplot2 (Wickham, 2016).

## 3 Results

### 3.1 Structural characterization of low- and high-molecular-weight MOS products

The carbohydrate composition of low- and high-molecular-weight manno-oligosaccharides (MOS) products is shown in Table 1. Low-molecular weight (LMW) MOS had a greater amount of mannobiose (14.6%) and mannose (32%) compared to HMW-MOS (3.6 and 24%, respectively) while HMW-MOS had a greater amount of oligomers in the DP 5–12 range (20%) compared to LMW-MOS (9%). Both LMW-MOS and HMW-MOS contain a small amount of proteins, lipids, and ash post-filtration.

**Table 1:** Carbohydrate composition of low- and high-molecular-weight manno-oligosaccharide products (LMW-MOS and HMW-MOS, respectively).

Component (% DM)	LMW-MOS	HMW-MOS
**DP 2–4 oligomers**	17.0	5.0
Mannobiose (DP 2)	14.6	3.6
Mannotriose (DP 3)	2.0	1.3
Mannotetraose (DP 4)	0.4	0.1
**DP 5–12**	9	20
**Mannose**	32	24
**Total monomers**	42	36

Concentrations show % dry matter (DM).

### 3.2 Growth inhibition assay of LMW-MOS, HMW-MOS, and high-purity MOS oligomers

Using the broth microdilution approach, we compared the growth of bacterial strains *E. coli K12* MG 1655, *K. pneumoniae* 1101160, *L. monocytogenes* 1071/53, and *S. mutans* NCTC 10449 with and without LMW-MOS, HMW-MOS, and mannose, using turbidity as a measure of bacterial growth. We selected *E. coli K12* as a model for type-1 fimbriated, mannose-mediated binding and *K. pneumoniae,* a fimbriated, highly virulent, and multidrug-resistant pathogen, in addition to two gram-positive pathogens *L. monocytogenes* and *S. mutans*.

**Figure 2:**
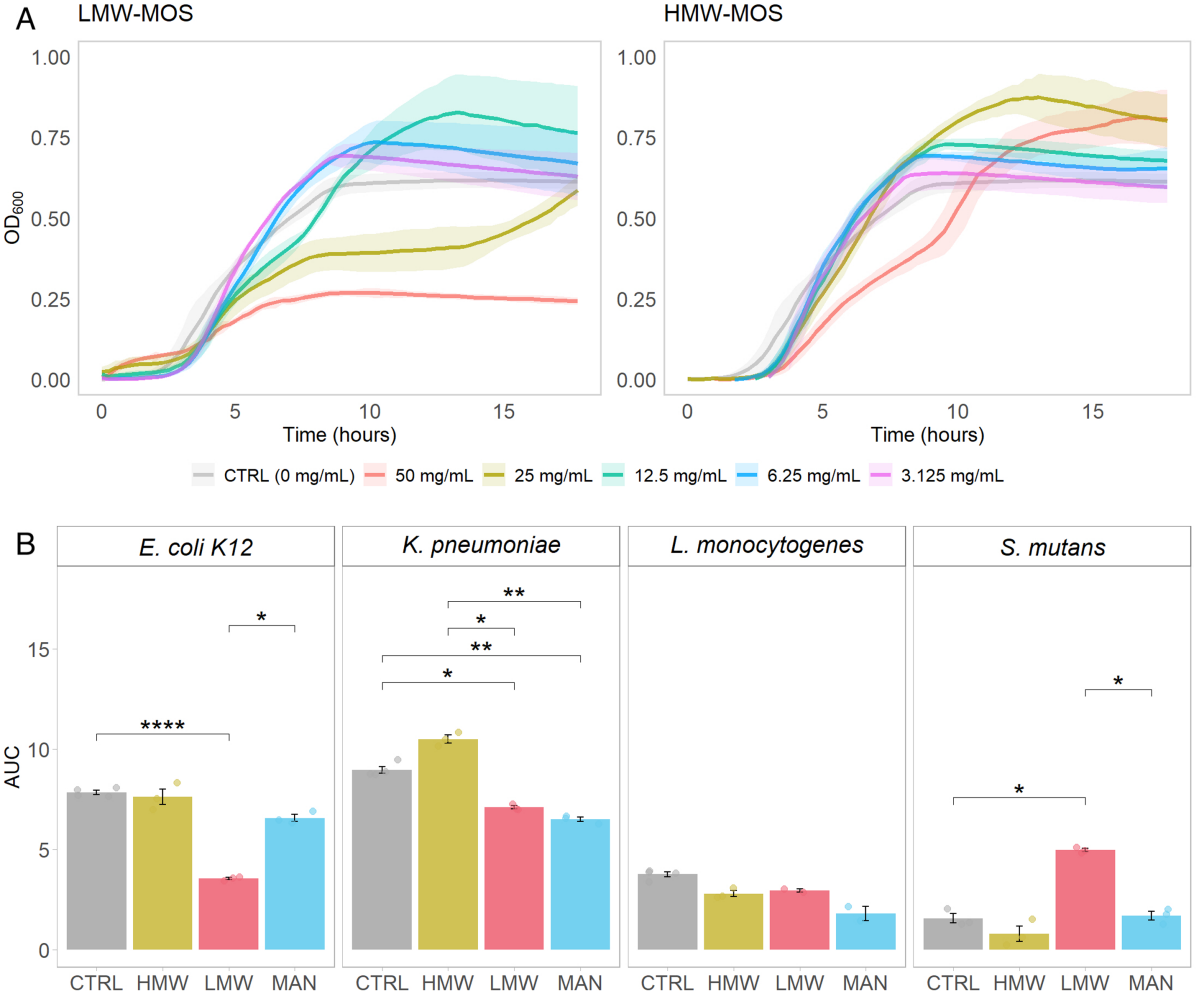
**(A)** Growth of *E. coli K12* growing on various concentrations of LMW-MOS (left) and HMW-MOS (right) over 18 h, showing dose–response. Shadowed error bars show standard error. **(B)** Area under the curve (AUC) of bacterial growth curves, as measured with OD_600_, of tested pathogens growing on LMW-MOS (50 mg/mL), HMW-MOS (50 mg/mL), and mannose (MAN, 50 mg/mL), representing cumulative growth. Error bars show standard error. Experimental AUC values were calculated using the R package growthcurver. Significance levels: no significant difference compared to the growth control (n.s.), significant at *p* > 0.05 (*), significant at *p* > 0.01 (**), significant at *p* > 0.001 (***), significant at *p* > 0.0001 (****). Significance was measured using Welch’s two-sample *t*-test.

In the first set of analyses, we compared the growth curves of *E. coli K12* growing on various concentrations of LMW-MOS and HMW-MOS (Figure 2A). Notably, 50 mg/mL of LMW-MOS inhibited the growth of *E. coli K12* compared to the control, while 25 mg/mL also inhibited growth but trended toward diauxic growth. Diauxic growth curves were also observed for *E. coli K12* cultures growing on 50 mg/mL of HMW-MOS, while HMW-MOS at concentrations of 25 mg/mL and below had greater growth compared to the media alone. Lower concentrations of both LMW-MOS and HMW-MOS increased growth compared to the growth control, rather than inhibiting growth.

After determining that the highest concentration tested (50 mg/mL) was the most inhibitory, we assessed the area under the curve (AUC) over the growth cycle for all strains via separate experiments with each of LMW-MOS, HMW-MOS, and mannose, at 50 mg/mL (Figure 2B). Compared to the growth control, 50 mg/mL LMW-MOS resulted in significantly lower AUCs for *E. coli* and *K. pneumoniae* and trended toward lower AUCs for *L. monocytogenes*. In contrast, 50 mg/mL HMW-MOS showed more variability and did not significantly decrease the AUCs for *E. coli K12*, *L. monocytogenes,* and *S. mutans*, but resulted in greater AUCs for *K. pneumoniae*. Notably, mannose at a concentration of 50 mg/mL inhibited the growth of *K. pneumoniae,* while not significantly impacting the growth of *E. coli K12* or *L. monocytogenes*. The full growth curves of all pathogens for all concentrations of LMW-MOS, HMW-MOS, and mannose are included in Supplementary files.

**Figure 3:**
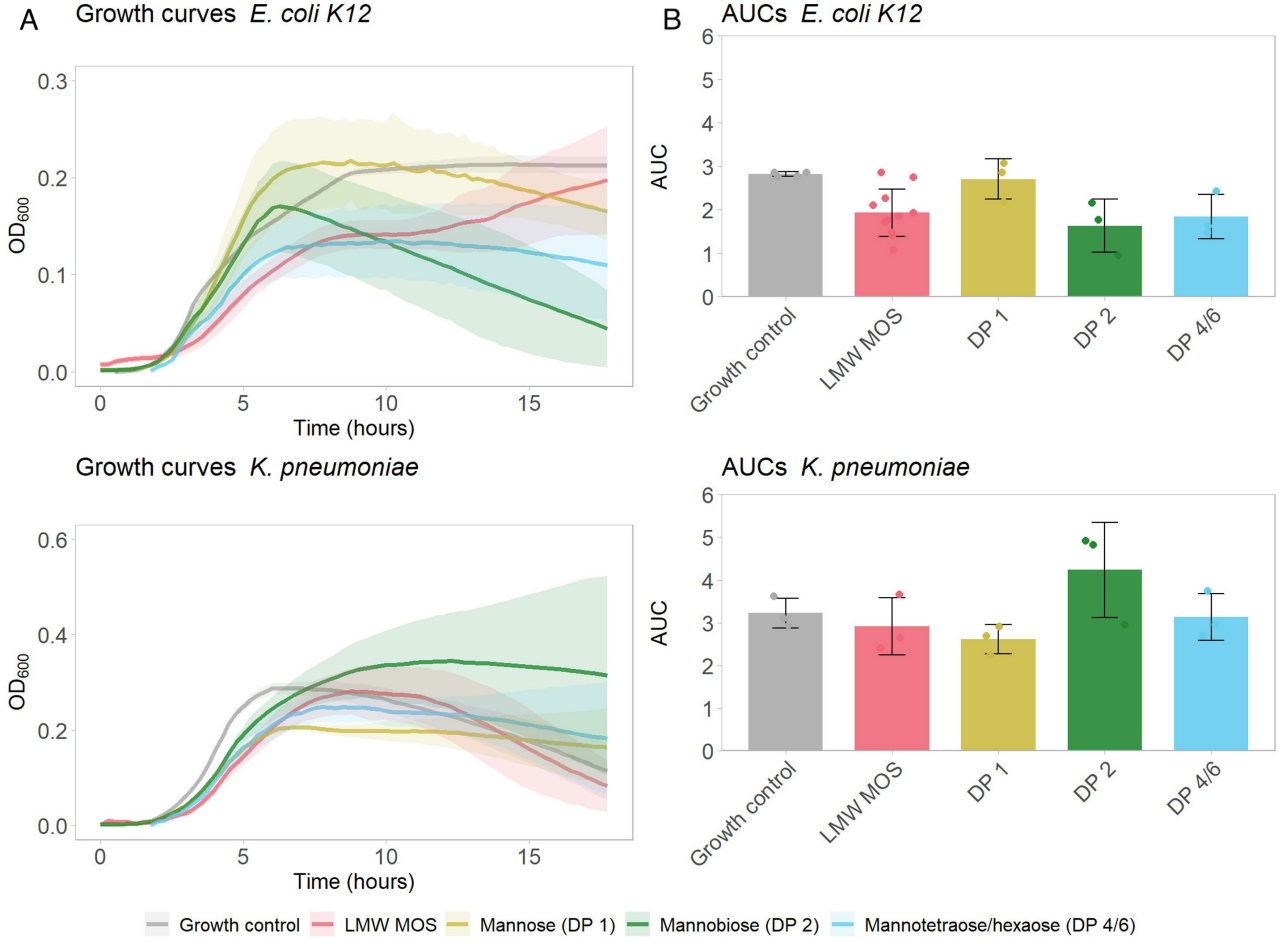
**(A)** Growth of *E. coli K12* (top) and *K. pneumoniae* (bottom) growing on various MOS products (each at a final concentration of 25 mg/mL), over 18 h. Shadowed error bars show standard error. **(B)** Area under the curve (AUC) of bacterial growth curves, as measured with OD_600_, of tested pathogens growing on LMW-MOS, HMW-MOS, and mannose, representing cumulative growth. Error bars show standard error. Experimental AUC values were calculated using the R package growthcurver.

Next, we aimed to determine how growth inhibition of type-1 fimbriated bacteria differed for distinct oligomer fractions. High-purity, HPLC-grade MOS oligomer products of DP 1 (mannose), DP 2 (mannobiose), and a 50:50 mixture of DP 4 and 6 (mannotetraose and mannohexaose) were used as substrates for cultures of *E. coli* and *K. pneumoniae* at concentrations of 25 mg/mL. Mannobiose and the mixture of mannotetraose and mannohexaose inhibited the growth of *E. coli* (Figure 3A), while mannobiose promoted the growth of *K. pneumoniae* (Figure 3B). Substrates LMW-MOS, mannose, and mannotetraose/hexaose trended toward lower AUCs for *K. pneumoniae* cultures than the growth control, and all substrates trended toward inhibiting the growth of *E. coli.*

### 3.3 Determination of MICs of antibiotics against bacterial strains

The broth microdilution method was used to determine the minimum inhibitory concentrations (MICs) of antibiotics against all pathogens before potentiation assays with MOS. The MICs are included in Table 2.

**Table 2:** Minimum inhibitory concentrations (MICs) of tested antibiotics against bacterial strains and sub-MICs, defined as half the MIC, and synergy with MOS.

Strain	Ceftazidime			Clindamycin			Gentamicin			Penicillin		
	MIC	Sub-MIC	Synergy with MOS*	MIC	Sub-MIC	Synergy with MOS*	MIC	Sub-MIC	Synergy with MOS*	MIC	Sub-MIC	Synergy with MOS*
*E. coli* K12 strain MG 1655	0.5	0.25	Promoted growth	N.S	N.S	–	16	8	Promoted growth	64	32	1x
*K. pneumoniae* strain 1101160	256	128	**2x**	128	64	>10x	64	32	–	N.S	N.S	0x
*L. monocytogenes* strain 1071/53	256	128	**>10x**	1	0.5	**>10x**	8	4	>10x	2	1	>10x
*S. mutans* strain NCTC 10449	0.5	0.25	**2x**	0.0156	0.0078	**5x**	8	4	1x	0.125	0.0625	Promoted growth

MICs and sub-MICs expressed as μg/mL. Antibiotics with no observed inhibition of bacteria are denoted by N.S. (Not Sensitive). *Synergy is defined as the reduction in final growth of bacterial cultures (OD_600_) exposed to 50 mg/mL MOS and a sub-MIC concentration of antibiotics compared to those with sub-MIC alone, with significant growth differences noted in bold.

### 3.4 Antibiotic potentiation by MOS

**Figure 4:**
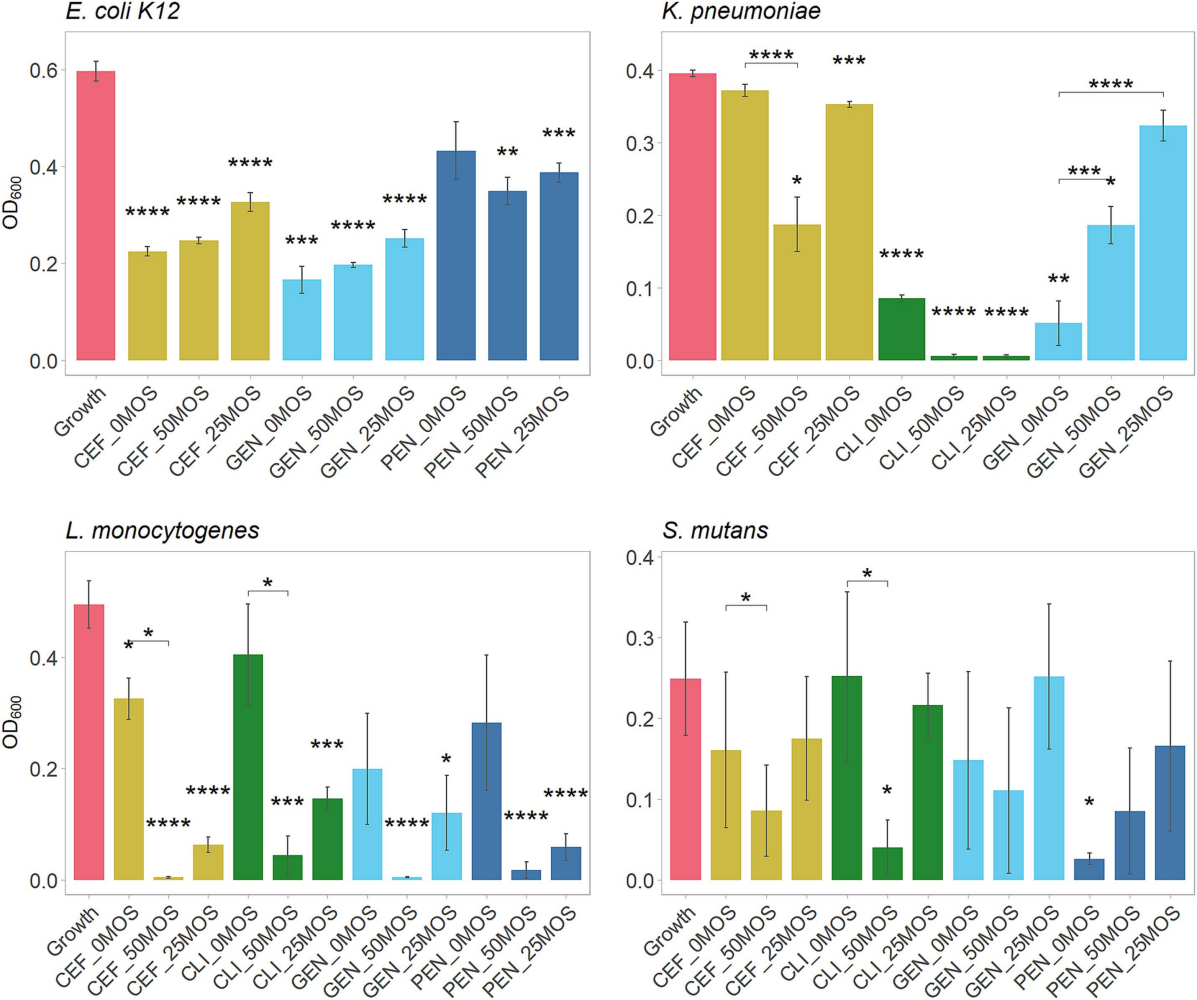
Antibiotic potentiation by MOS, showing growth of pathogens alone (growth control) and pathogens after 24 h with a combination of either 50 or 25 mg/mL of LMW-MOS, or the antibiotic alone (PBS alone). Bacterial growth measured at OD_600_ after 24 h. Tested antibiotics included ceftazidime (CEF), clindamycin (CLI), gentamicin (GEN), and penicillin (PEN). Assays performed on three separate days (biological replicates) using a minimum of two technical replicates each. Asterisks above bars indicate significance compared to the growth control (no antibiotic and no MOS), and asterisks between bars indicate significance compared to the antibiotic alone (no MOS). Significance was measured using independent *t*-tests, with *p*-values adjusted for multiple comparisons using Bonferroni *post-hoc* tests. Error bars show standard error. Significance levels: * showing p.adj < 0.05, ** showing p.adj < 0.01, *** showing p.adj < 0.001, **** showing p.adj < 0.0001.

Next, we were interested in studying whether MOS can potentiate the action of antibiotics against the studied bacterial strains. A sub-MIC concentration was selected to determine what concentration of MOS, if any, would result in lower bacterial growth than a sub-MIC concentration of the antibiotic alone (Figure 4). A summary of the synergistic effects of 50 mg/mL MOS with tested antibiotics against pathogens is included in Table 2.

Against *E. coli*, only penicillin demonstrated trends of potentiation by MOS, while MOS promoted the growth of cultures with ceftazidime and gentamicin in a dose-dependent manner. Both 50 mg/mL and 25 mg/mL MOS potentiated clindamycin and ceftazidime against *K. pneumoniae*, resulting in very little final bacterial growth with clindamycin and half as much growth by 50 mg/mL MOS with ceftazidime (final OD_600_ of 0.19 [SE: 0.03, 0.35] compared to 0.37 [0.34, 0.41], p.adj < 0.0001). All antibiotics against *L. monocytogenes* trended toward being potentiated by MOS in a dose-dependent manner, with 50 mg/mL MOS significantly potentiating ceftazidime (final OD_600_ of 0.01 [−0.01, 0.01] compared to 0.33 [0.16, 0.49], p.adj < 0.0001) and clindamycin (final OD_600_ of 0.04 [−0.01, 0.19] compared to 0.41 [0.01, 0.79], p.adj < 0.001). The addition of 50 mg/mL MOS potentiated the action of clindamycin against *S. mutans* and, to a lesser degree, ceftazidime. In some cases, however, antibiotics were rendered less effective by the addition of MOS to the media; for example, both 50 mg/mL and 25 mg/mL MOS reduced the effectiveness of gentamicin against *K. pneumoniae*.

### 3.5 Bradford assay to measure protein contents in culture supernatant

**Figure 5:**
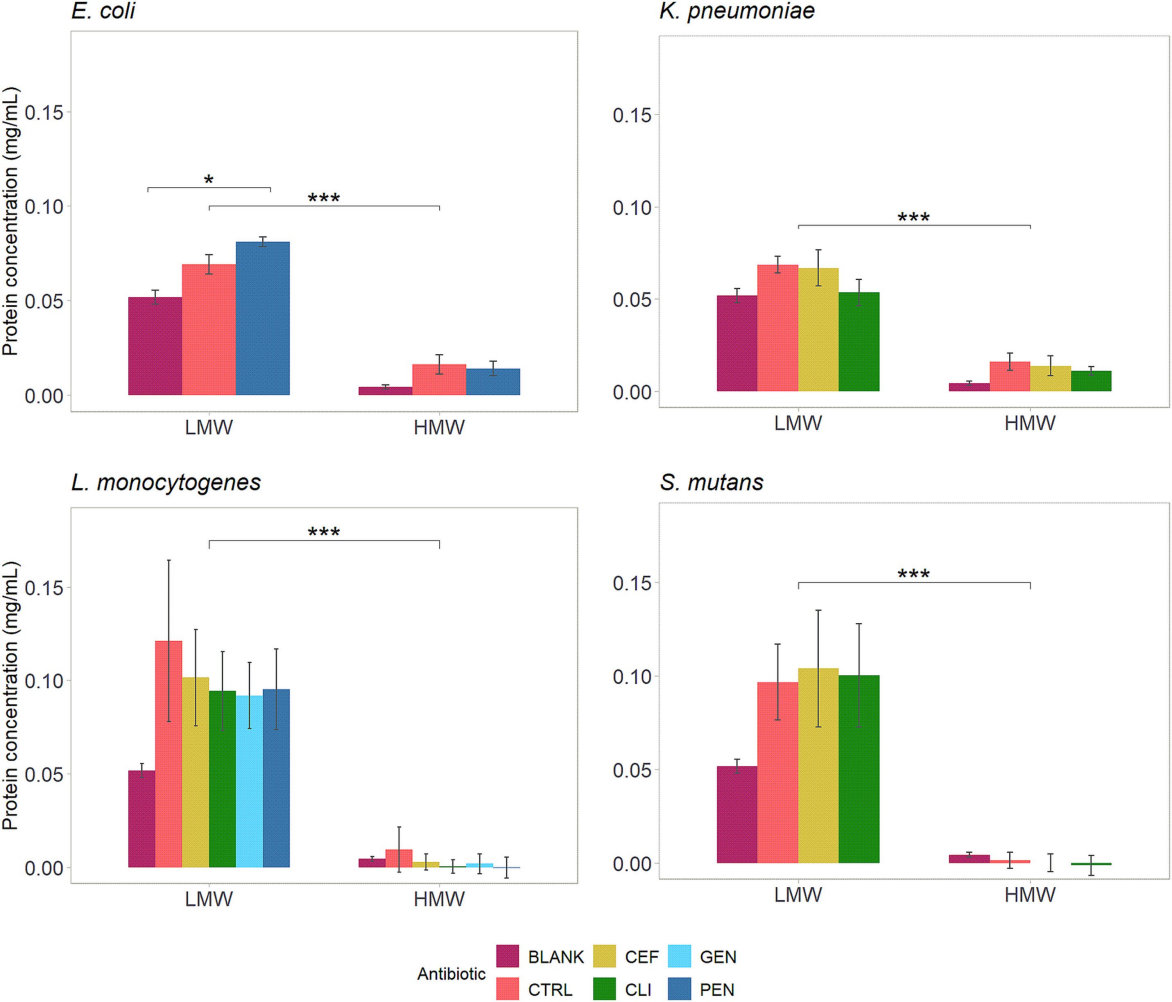
Effect of 25 mg/mL MOS and antibiotics on protein concentrations in the supernatant of bacterial culture following 1 h of incubation. Assays performed on three separate days (biological replicates) using a minimum of two technical replicates each. Blank values represent the protein concentration in the MOS alone, without culture added. Control represents the protein concentration in the culture with MOS alone, without antibiotics. The significance between MOS types (LMW vs. HMW) and between antibiotic conditions was assessed using independent *t*-tests, with *p*-values adjusted for multiple comparisons using Bonferroni *post-hoc* tests. Significance levels: * shows p.adj < 0.05, ** shows p.adj < 0.01, *** shows p.adj < 0.001, and **** shows p.adj < 0.0001. Error bars show standard error.

The total protein concentration in the supernatant of cultures was measured after 1 h of incubation with LMW-MOS or HMW-MOS in combination with antibiotics, to explore possible mechanisms of inhibition and antibiotic potentiation (Figure 5). Significant differences in the relative increase in protein concentration were observed across cultures for both MOS types. While LMW-MOS had a greater protein content at baseline, as evidenced by the comparison of each growth control (CTRL) to the blank condition (BLANK), cultures grown with LMW-MOS had a greater amount of protein released into the supernatant than those grown with HMW-MOS, especially for gram-positive pathogens *L. monocytogenes* and *S. mutans*.

The protein concentration was also assessed in cultures incubated with both types of MOS and antibiotics (Figure 5). Cultures of *E. coli* incubated with penicillin and LMW-MOS had a statistically significant increase in protein concentration compared to LMW-MOS alone. For all other pathogen-antibiotic conditions, incubating bacterial cultures with MOS and antibiotics resulted in lower protein concentrations in the supernatant than incubating cultures with MOS alone.

## 4 Discussion

In this study, we explored various anti-bacterial properties of mannose-based oligomers with strains *E. coli K12* MG 1655, *K. pneumoniae* 1101160, *L. monocytogenes* 1071/53, and *S. mutans* NCTC 10449. These bacteria were selected to explore a range of anti-pathogenic mechanisms that might be exhibited by manno-oligosaccharides (MOS), such as mannose-mediated binding of fimbriated *E. coli K12* and *K. pneumoniae* and the ability of MOS to potentiate antibiotics against these bacteria in addition to gram-positive pathogens *L. monocytogenes* and *S. mutans*.

We tested two MOS products from copra meal and varied the process parameters to produce a higher-molecular-weight product (HMW-MOS) and a lower-molecular-weight product (LMW-MOS). These MOS products, along with high-purity individual MOS fractions, were used to determine the impact of carbohydrate structure on the inhibitory ability of MOS. Notably, differences were seen in the inhibition profiles of the structurally distinct LMW-MOS and HMW-MOS: *E. coli K12* was inhibited by both LMW-MOS and HMW-MOS, while cultures of *K. pneumoniae* were inhibited only by LMW-MOS. Although both LMW-MOS and HMW-MOS are composed of mannose-based oligomers, LMW-MOS had a higher proportion of oligomers in the DP 2–4 range (17% compared to 5% for HMW-MOS), whereas HMW-MOS had more oligomers in the DP 5–12 range (20% compared to 9% for LMW-MOS). These findings suggest that even small differences in the structure and degree of polymerization of oligosaccharides can have significant differences in their biological effects.

The difference in the inhibition profiles between LMW-MOS and HMW-MOS led us to hypothesize that different oligomer fractions within the MOS mixture were responsible for different inhibition abilities. We aimed to explore this further by repeating the assays of *E. coli K12* and *K. pneumoniae* cultures with individual MOS oligomers mannobiose (DP 2) and a 50/50 combination of mannotetraose (DP 4) and mannohexaose (DP 6). Notably, distinct growth curves were observed for each of these oligomer fractions: while cultures of *E. coli K12* showed the greatest decrease in growth when grown with mannobiose, the growth of *K. pneumoniae* cultures was suppressed only by mannose (DP 1). These findings suggest that *K. pneumoniae* is inhibited more by mannose than by mannose-based oligomers, aligning with the greater inhibition observed by LMW-MOS, which is considerably higher in mannose than HMW-MOS (32% vs. 8% DM). On the other hand, *E. coli K12* appears to be inhibited more by short-chain oligomers, such as DP 2-6, and little inhibition is observed with mannose alone. The differences in inhibition may be related to the differences in binding between fimbriae and mannose: while it is generally accepted that the majority of type-1 fimbriae bind trisaccharide oligo-mannose compounds, only some *E. coli* and *K. pneumoniae* have a genetic mutation that more tightly binds mannose monomers (Stahlhut et al., 2009).

In our study, high concentrations of MOS inhibited bacterial growth, while lower concentrations could promote growth. We reported the growth curves for *E. coli* cultures growing on MOS of concentrations between 3.1 and 50 mg/mL, and this trend was observed for all four bacterial strains we tested (full growth curves for all cultures are included in Supplementary material). Other studies have reported inhibition of pathogens by oligosaccharides only at higher concentrations, such as for *S. aureus* and *P. aeruginosa* with an alginate oligosaccharide (Jack et al., 2019) and *S. aureus* and *S. agalactiae* with chito-oligosaccharides (Asadpoor et al., 2021a). One likely explanation for this is that even high-purity oligosaccharide products have small amounts of monomers that may be utilized by microbes for cell growth. It is likely that the growth at lower concentrations is a result of these cultures utilizing monomers or other oligomeric components within the product composition for growth when the concentration of MOS is below that required for inhibition. This effect may also explain the diauxic growth curves observed in our study for intermediate concentrations of LMW-MOS (25 and 12.5 mg/mL) and the highest concentration of HMW-MOS (50 mg/mL). In addition, it should be noted that similar trends have been observed for other compounds with anti-bacterial effects: even antibiotics can, in some cases, stimulate growth at sub-lethal concentrations (Agathokleous et al., 2018). Oligosaccharides composed of mannose sub-units may offer the additional advantage of being utilized to a lesser degree than those that are fructan- or glucan-based. The widespread presence of fructofuranosidases and galactosidases in bacteria, including pathogenic bacteria, can lead to significant growth of pathogens on other prebiotics. In one study measuring bacterial growth on various prebiotic substrates, GOS and FOS promoted the growth of pathogens such as *E. coli* EHEC and *C. perfringens,* while various XOS products inhibited growth (Mäkeläinen et al., 2010; Saville and Saville, 2020).

In addition to mannose-mediated binding, other mechanisms have been proposed for how mannose and MOS inhibit bacterial growth. One study demonstrated that mannose-containing oligosaccharides in the glycated secretory component of secretory IgA mediated the inhibition of biofilm formation of *Vibrio cholerae* (Murthy et al., 2011). Further experiments showed that biofilm formation could be inhibited by mannose in a dose-dependent manner (Murthy et al., 2011), demonstrating the importance of mannose for antimicrobial effects against a range of bacteria. In this study, we found that *L. monocytogenes* and *S. mutans* trended toward inhibition by mannose and MOS, but with more subtle effects than for the inhibition of *E. coli K12* and *K. pneumoniae*.

One interesting property reported for some oligosaccharides is their ability to potentiate the action of antibiotics, rendering antibiotics more effective against a target pathogen. Protein synthesis-inhibiting clindamycin and gentamicin, and cell wall synthesis-inhibiting penicillin and ceftazidime were selected to explore synergistic effects with MOS. One notable finding was the ability of 50 mg/mL LMW-MOS to render ceftazidime more effective against *K. pneumoniae*, a pathogen with increasing resistance to antibiotics. Other promising MOS-antibiotic pairs include *L. monocytogenes* with ceftazidime and clindamycin, as well as *E. coli K12* with penicillin. For all antibiotic-pathogen pairs, the higher concentration of MOS showed the strongest potentiation abilities. One proposed mechanism for the ability of oligosaccharides such as MOS to potentiate antibiotics is by disrupting biofilm formation, which allows antibiotics greater access to free, planktonic bacteria (Asadpoor et al., 2021a). Of the pathogens tested, *S. mutans*, *K. pneumoniae*, and *L. monocytogenes* are known to form biofilms. Oligosaccharides have also been reported to interact directly with bacterial cell membranes: in one study, charged alginate oligosaccharides directly interacted with the cell membrane of gram-negative *P. aeruginosa*, reducing motility and disrupting biofilm formation (Powell et al., 2014). Other studies demonstrate that exposure of bacteria to some oligosaccharides resulted in an increase in nucleic acids and proteins in the culturing media, possibly as an effect of increasing bacterial membrane permeability (Lu et al., 2019). In another study, cultures of Group-B *Streptococcus* (GBS) exposed to HMOs had altered fatty acid and lipid metabolism, resulting in the accumulation of metabolites that are related to cell membrane construction (Chambers et al., 2020). The same group found that HMOs potentiated the action of antibiotics and theorized that the mechanism by which HMOs increase the effectiveness of these antibiotics is related to increased membrane permeability (Craft et al., 2018b).

To explore potential mechanisms of how MOS potentiated the action of antibiotics, we incubated LMW-MOS and HMW-MOS with bacteria cultures and evaluated whether MOS increased protein in the culture media, which would be consistent with an increase in membrane permeability. We found that LMW-MOS had a much greater impact on increasing the protein content in the supernatant of cultures than HMW-MOS, a trend that was seen across cultures of all four bacterial strains. This difference may be related to the higher proportion of smaller, more soluble oligomer fractions in the LMW-MOS, which can more readily interact with bacterial cell membranes and are more easily transported into cells. We also measured the difference in protein concentration within cultures incubated with both MOS and antibiotics and found that *E. coli* incubated with both penicillin and LMW-MOS had a statistically significant increase in protein concentration compared to LMW-MOS alone. For all other pathogen-antibiotic conditions, incubating bacterial cultures with MOS and antibiotics resulted in no significant difference in protein concentration, and, in some cases, there was a trend to lower extracellular protein concentrations.

While these study findings suggest that MOS, particularly LMW-MOS, exhibits abilities to inhibit bacterial growth and increase bacterial membrane permeability, there are limitations to this research. First, we used a turbidimetric approach to measure growth inhibition, hypothesizing that mannose and mannose oligomers would bind to fimbriae of bacterial cells growing within the liquid medium, preventing mobility and subsequently growth, in line with other literature exploring growth inhibition of mannose and mannose derivatives (Shubhashini et al., 2022; Srivastava et al., 2017). However, it is possible that fimbriated enterobacteria can be bound by mannose compounds while still being able to grow, as has been reported by other authors: in one study, yeast-based *α*-MOS (technically, mannans based on reported molecular weights and solubility) promoted the growth of uropathogenic *E. coli* (UPEC) but inhibited its adhesion to uroepithelial cells (Faustino et al., 2023). In addition, while we demonstrated that bacterial cultures growing with LMW-MOS had a greater concentration of protein in the culture supernatant, which we hypothesize is related to leakage of intracellular protein contents, further research is required to confirm mechanistic effects and whether LMW-MOS induces membrane disruption or induces other cellular processes that contribute to protein release.

It is clear that MOS can inhibit the growth of fimbriated bacteria *in vitro* and that inhibition effects are distinct, depending on the structure of MOS. This research aligns with a wide body of evidence showing that mannose and mannose-based oligomers interact with pathogens and inhibit their growth. Compared to conventional antibiotics, MOS do not have the same risk of promoting antibiotic resistance, being bacteriostatic rather than bactericidal, and may further promote intestinal health through their prebiotic role in intestinal microbial communities.

## 5 Conclusion

In this study, we demonstrate the ability of soluble beta-linked manno-oligosaccharides (MOS) from coconut to inhibit the growth of various pathogenic bacteria *in vitro* and confirm that carbohydrate structure influences pathogen inhibition. Notably, distinct MOS fractions differentially inhibited the growth of cultures of type-1 fimbriated *E. coli* and *K. pneumoniae*. Low-molecular weight MOS potentiated the action of ceftazidime and clindamycin against multidrug-resistant *K. pneumoniae*, among other pathogens, and was associated with a greater release of protein into the supernatant of bacteria cultures. Furthermore, these findings demonstrate the importance of dose when considering oligosaccharides with inhibitory or anti-bacterial properties. To the best of our knowledge, this is the first report on the ability of plant-derived mannose-based oligosaccharides to potentiate antibiotics. These findings highlight the potential for soluble beta-linked MOS to be used to mediate bacterial infections as a lower-risk and cost-effective alternative to antibiotics. Further research should be conducted to explore possible mechanisms of MOS and mannose derivatives for pathogen growth inhibition and antibiotic potentiation.

## Data Availability

The datasets generated for this study can be found in Borealis, the Canadian Dataverse Repository at: https://doi.org/10.5683/SP3/JC4Y5A.
