# Supplementary material for: Manno-oligosaccharides as a promising antimicrobial strategy: pathogen inhibition and synergistic effects with antibiotics
Source: Front Microbiol. 2025 Mar 24;16:1529081. doi: 10.3389/fmicb.2025.1529081 (PMC11973258; doi:10.3389/fmicb.2025.1529081)
Supplement: Supplementary file 1 [file Data_Sheet_1.docx]

**Supplementary Materials**

| Substrate [mg/mL] | 50 | 25 | 12.5 | 6.25 | 3.13 | 1.56 | 0.78 | 0.39 | 0.20 | 0.10 | Growth control | Sterility controls |
| --- | --- | --- | --- | --- | --- | --- | --- | --- | --- | --- | --- | --- |
|  | 1 | 2 | 3 | 4 | 5 | 6 | 7 | 8 | 9 | 10 | 11 | 12 |
| A | Pathogen #1 |  |  |  |  |  |  |  |  |  |  | Broth sterility control |
| B |  |  |  |  |  |  |  |  |  |  |  |  |
| C |  |  |  |  |  |  |  |  |  |  |  |  |
| D | Pathogen #2 |  |  |  |  |  |  |  |  |  |  | MOS sterility control |
| E |  |  |  |  |  |  |  |  |  |  |  |  |
| F |  |  |  |  |  |  |  |  |  |  |  |  |
| G |  |  |  |  |  |  |  |  |  |  |  |  |
| H |  |  |  |  |  |  |  |  |  |  |  |  |

Table: Example of 96-well plate for Minimum Inhibitory Concentration (MIC) assays and growth inhibition assays. Each pathogen tested in duplicate or triplicate, i.e., in rows A, B or rows A, B, C.


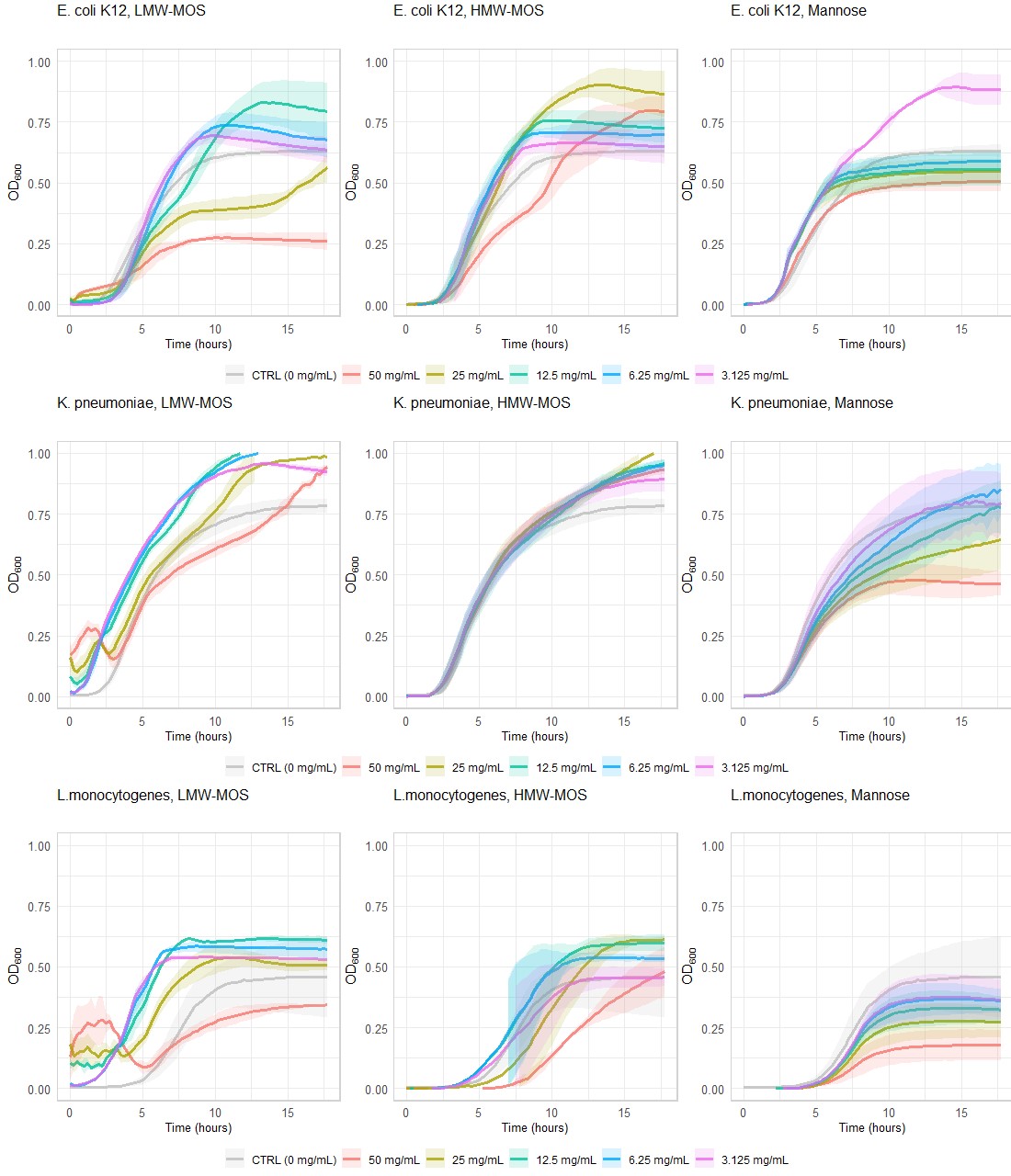


**Figure:** Growth curves for *E. coli K12*, *K. pneumoniae*, and *L. monocytogenes* growing on LMW-MOS, HMW-MOS, and mannose.
